# Development and Recovery of Liver Injury in Piglets by Incremental Injection of LPS

**DOI:** 10.3390/antiox12061143

**Published:** 2023-05-24

**Authors:** Geyan Duan, Pan Huang, Changbing Zheng, Jie Zheng, Jiayi Yu, Peiwen Zhang, Mengliao Wan, Fengna Li, Qiuping Guo, Yulong Yin, Yehui Duan

**Affiliations:** 1 CAS Key Laboratory of Agro-Ecological Processes in Subtropical Region, Hunan Provincial Key Laboratory of Animal Nutritional Physiology and Metabolic Process, National Engineering Laboratory for Pollution Control and Waste Utilization in Livestock and Poultry Production, Institute of Subtropical Agriculture, Chinese Academy of Sciences, Scientific Observing and Experimental Station of Animal Nutrition and Feed Science in South-Central, Ministry of Agriculture, Changsha 410125, China; duangeyan21@mails.ucas.ac.cn (G.D.); huangpan@isa.ac.cn (P.H.); zhengjie202@mails.ucas.ac.cn (J.Z.); yujiayi22@mails.ucas.ac.cn (J.Y.); lifengna@isa.ac.cn (F.L.); guoqiuping@isa.ac.cn (Q.G.); yinyulong@isa.ac.cn (Y.Y.); 2 University of Chinese Academy of Sciences, Beijing 100049, China; 3 College of Animal Science and Technology, Hunan Agricultural University, Changsha 410128, China; chamdpion@stu.hunau.edu.cn (C.Z.); 1228065671@stu.hunau.edu.cn (P.Z.); wmengliao01@stu.hunau.edu.cn (M.W.)

**Keywords:** weaned piglets, liver injury and restoration, lipopolysaccharide

## Abstract

This study aimed to explore the effects of the incremental injection of lipopolysaccharide (LPS) on liver histopathology, inflammation, oxidative status, and mitochondrial function in piglets. Forty healthy Duroc × Landrace × Yorkshire castrated boars (21 ± 2 days old, weight 6.84 ± 0.11 kg) were randomly assigned to five groups (*n* = 8) and then slaughtered on days 0 (group 0, without LPS injection), 1 (group 1), 5 (group 5), 9 (group 9), and 15 (group 15) of LPS injection, respectively. The results showed that, compared to the piglets without LPS injection, LPS injection caused liver injury in the early phase, as manifested by the increased activities of serum liver injury-related parameters (aspartate amino transferase, alanine aminotransferase, alkaline phosphatase, cholinesterase, and total bile acid) on day 1, and impaired liver morphology (disordered hepatic cell cord arrangement, dissolved and vacuolized hepatocytes, karyopycnosis, and inflammatory cell infiltration and congestion) on days 1 and 5. Meanwhile, LPS injection caused liver inflammation, oxidative stress, and mitochondrial dysfunction on days 1 and 5, as reflected by the upregulated mRNA expression of TNF-α, IL-6, IL-1β, TLR4, MyD88, and NF-κB; increased MPO and MDA content; and impaired mitochondrial morphology. However, these parameters were ameliorated in the later phase (days 9~15). Taken together, our data indicate that the incremental injection of the LPS-induced liver injury of piglets could be self-repaired.

## 1. Introduction

To enhance productive efficiency, modern swine production is becoming increasingly intensive, which augments the exposure risk of weaned piglets to many stressful events, especially in terms of immune stress [1,2]. In the early period after weaning, this stress occurs frequently and impairs pig growth and health, ultimately causing immeasurable economic losses to the livestock industry [3,4]. A current area of interest in the field is how intestinal injury occurs and which nutrients are regulatory [5,6,7]. Apart from the intestine, another major area of emerging interest is the impact of immune stress on the liver, since it is the final barrier that protects the body from the effects of exogenous toxic substances [8]. Over the past few years, there has been an explosion in knowledge about the role of nutrients in liver injury induced by immune stress [9,10,11,12,13,14,15,16]. Findings from these studies advance the understanding of the amelioration of immune stress-induced liver injury. It is of note that much of the abovementioned work has been carried out on swine models regarding short term and acute stress. However, it is chronic but not acute immune stress that is more common in the actual pig farm environment, which is often accompanied by bacterial proliferation. Therefore, to pave the way for the therapeutic intervention of liver injury induced by chronic immune stress, a better understanding of how chronic immune stress and liver injury are bridged is a task which brooks no delay.

Physiologically, the liver and intestine are in extensive communication via the portal vein, biliary tract, and systemic circulation, making the liver vulnerable to gut-derived toxins, such as bacteria and bacterial products [8]. Among them, lipopolysaccharide (LPS), the major constituent of the outer membrane of Gram-negative bacteria, can stimulate Kupffer cells (resident macrophages in the liver) to release various inflammatory cytokines (e.g., interleukin-6 (IL-6), interleukin-1β (IL-1β), and tumor necrosis factor-α (TNF-α)) and cytotoxic mediators (e.g., reactive oxygen species (ROS)) [17]. When these mediators are continuously produced and are beyond the tolerance range of the liver, inflammatory liver injury occurs [18,19]. In addition, ROS-triggered oxidative stress has been reported to be accompanied by ultrastructural damage with mitochondria and mitochondrial dysfunction (e.g., impaired mitochondrial biogenesis and oxidative phosphorylation), thus exacerbating liver injury [20]. Meanwhile, to reduce oxidative stress caused by ROS, cells express antioxidants and ROS scavengers, including superoxide dismutase (SOD, including copper and zinc superoxide dismutase (Cu/Zn-SOD) and manganese-containing superoxide dismutase (Mn-SOD), glutathione peroxidase 1 (GPx-1/GSH-Px), thioredoxin reductase 1 (TXNRD), heme oxygenase 1 (HMOX1), myeloperoxidase (MPO), and malondialdehyde (MDA) [21,22,23,24,25]. However, to date, little is known about the effects of the incremental injection of LPS-induced chronic immune stress on inflammation, mitochondrial function, and oxidative status in the liver of piglets or other species. Therefore, further research is warranted.

Animal immune stress models are often constructed via the intraperitoneal injection of LPS [26]. However, in the acute immune stress model induced through a single injection of a certain dose of LPS, there are several shortcomings, such as susceptibility to immune tolerance and short duration. To mimic the immune stress existing in the actual pig farm, researchers have built a chronic immune stress model by injecting LPS incrementally every other day, which has been shown to lead to systemic inflammation and damage to the liver, intestine, muscle, and other tissues and organs [27,28,29]. Therefore, this experiment aimed to explore the effects of chronic immune stress established by injecting LPS incrementally every other day on inflammation, mitochondrial function, and oxidative status in the livers of piglets.

## 2. Materials and Methods

### 2.1. Animals and Diets

Animal protocols complied with the accepted animal care standards and were approved by the Animal Care and Use Committee of the Institute of Subtropical Agriculture, Chinese Academy of Sciences (Changsha, China, ISA-2020-0005).

**Figure 1:**
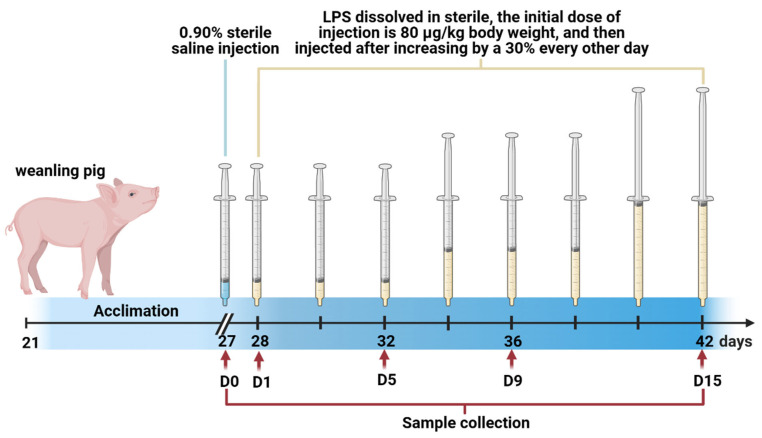
The scheme of the experimental design. After a one-week acclimation period, forty 21-day-old pigs were randomly allotted to five groups with eight replicates per treatment: (1) day 0 group (piglets were injected with 0.90% sterile saline and slaughtered before day 1 of the trial); (2) piglets in the day 1, day 5, day 9, or day 15 groups were injected with LPS dissolved in sterile media at an initial dose of 80 μg/kg body weight, were then injected with an increase of 30% every other day, and were slaughtered on day 1, day 5, day 9, and day 15, respectively.

After a one-week acclimation period, forty healthy piglets that were weaned at 21 days (6.84 ± 0.11 kg, Duroc × Landrace × Yorkshire, castrated boars) were chosen and randomly assigned to five groups with eight replicates per treatment. Piglets were slaughtered on days 0 (0 group, without LPS injection), 1 (1 group), 5 (5 group), 9 (9 group), and 15 (15 group) of LPS injection, respectively. From day 1 of the trial, piglets were injected intraperitoneally with LPS (E. coli serotype 055: B5; Sigma Chemical, St Louis, MO, USA), the injection dose was the same as what we previously reported [5]. The same amount of 0.90% sterile saline was injected into piglets in the 0 group, which were slaughtered 1 day before the trial. The scheme of the experimental design is shown in Figure 1. During the experimental period of 15 days, the piglets were kept in 1.80 × 1.10 m cages separately (their temperatures remained constant at 25–27 °C) and were allowed *ad libitum* access to food and water. All diets were formulated to meet the nutrient requirements and physiological needs of weaned piglets, and the ingredients and nutritional values are given in our previous studies [5].

### 2.2. Sample Collection

On days 0, 1, 5, 9, and 15 of the trial, blood samples were taken from the anterior vena cava via venipuncture at three hours after the intraperitoneal injection of saline or LPS and then centrifuged at 4 °C and 3000× *g* for 15 min to separate the serum. Then, the supernatant was stored in a refrigerator at −80 °C for the detection of serum biochemical parameters. Next, all piglets were stunned with an electric current (250 V, 0.5 A) for 6 s after blood collection for 3 h and then slaughtered. Then, the liver samples were rapidly excised from the fixed position of the liver. A portion of the liver samples was deposited in 4% paraformaldehyde solution for liver morphological analysis or in 2.5% glutaraldehyde solution for the observation of liver mitochondrial ultrastructure; the other portions were placed in liquid nitrogen as soon as possible and stored at −80 °C until further analysis.

### 2.3. Serum Biochemical Indexes 

Corresponding commercial kits, purchased from Sino-German Beijing Leadman Biotech Ltd. (Beijing, China), and an automatic biochemistry analyzer (Roche, Basel, Switzerland) instrument were used to measure the concentrations of serum alkaline phosphatase (ALP), alanine aminotransferase (ALT), aspartate amino transferase (AST), cholinesterase (CHE2), and total bile acid (TBA). 

### 2.4. Histopathological Evaluation

Liver tissue was deposited into 4% paraformaldehyde, dehydrated, and then embedded in paraffin. Then, for different histological examinations, the hydrated tissue sections were either stained with hematoxylin and eosin (H&E) or Masson–Trichrome, as previously described [16,30].

### 2.5. Redox Parameters, ROS, and Mitochondrial Membrane Potential Detection

A 10% liver tissue homogenate was prepared and used to detect the redox parameters (including total antioxidant capacity (T-AOC), the superoxide dismutase (SOD), glutathione peroxidases (GSH-Px), myeloperoxidase (MPO), and malondialdehyde (MDA)) by using their corresponding porcine-specific kits obtained from Nanjing Jiancheng Bioengineering Institute (Nanjing, China). Results are shown by enzyme activity.

A cell mitochondrial isolation kit (SM0020) brought from Beijing Solarbio Science & Technology Co., Ltd. (Beijing, China), was used to extract mitochondria from fresh liver tissues, as previously described [31]. Then, the extracted mitochondria was used to detect the mitochondrial membrane potential (CA1310, Solarbio, Beijing, China) and ROS content (CA1410, Solarbio, Beijing, China), according to the corresponding manufacturer’s protocols. The green and red fluorescence calculated by Image J V1.8.0 software indicate the apoptotic and normal conditions of cells, respectively. The fluorescence intensity of ROS content was determined through flow cytometry (BD, FACSCalibur, Becton Dickinson, Franklin Lakes, NJ, USA) [32].

### 2.6. Reverse Transcription and Quantitative Real-Time PCR

According to the methods we previously reported, the reverse transcription and quantitative real-time PCR of liver tissues were performed [33]. The primer sequences for the target genes are listed in Table 1. The 2^−ΔΔCt^ method was used to calculate the relative mRNA expression levels of the target genes, with β-actin as the endogenous reference gene.

### 2.7. Immunofluorescence

The expression levels of hepatic CD3 (T-cell marker) and CD68 (macrophage-specific marker) proteins were analyzed via immunofluorescent staining. The fluorescence intensity of CD3 and CD68 proteins were quantified by Image J (NIH, Bethesda, MD, USA). Analysis was carried out on ten randomly chosen fields in each section.

### 2.8. Mitochondrial Ultrastructure

The pruned treated liver tissue block was fixed using 2.5% glutaraldehyde and then used to detect the mitochondrial ultrastructure by using the transmission electron microscopy, as previously reported [5]. 

### 2.9. Statistical Analysis

All data were analyzed using SAS 8.2 software (Institute, Inc., Cary, NC, USA). Significant differences among different treatments were analyzed via one-way ANOVA, followed by Tukey’s HSD comparisons. According to the report [34], all data were tested for normality through the Shapiro–Wilk test and were found to be normal. The Levene test was used for the homoscedasticity test. The Welch test analysis was used when the data did not meet the homogeneity of variance, and the box plot text was used to distinguish the data. All data were expressed in mean ± standard error. The difference in *p* < 0.05 was statistically significant. 

## 3. Results

### 3.1. Serum Biochemical Indexes

**Figure 2:**
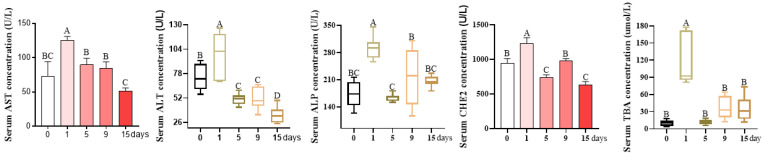
LPS-induced serum biochemical parameters, referring to liver injury. Day 0 (control group): pigs were injected with 0.90% sterile saline; pigs in the day 1, 5, 9, or 15 groups were injected with LPS dissolved in sterile saline at an initial dose of 80 μg/kg body weight and then injected with an increase of 30% every other day. AST, aspartate amino transferase; ALT, alanine phosphatase; ALP, alkaline phosphatase; CHE2, cholinesterase 2; TBA, total bile acid. Values are the mean ± SEM (*n* = 8). The row values of different capital letters A, B, C, and D were significantly different (*p* < 0.05). Data represented by the column bar graph were tested via one-way ANOVA, and data represented by the box plot were evaluated via Welch’s test.

As revealed in Figure 2, compared to the day 0 group, the serum activities of AST, ALT, ALP, CHE2, and TBA were significantly increased by 72.80%, 30.50%, 68.62%, 30.54%, and 1069.23%, respectively, on day 1, and had similar values on days 5, 9, and 15 (*p* < 0.05).

### 3.2. Histopathological Observation of Livers

**Figure 3:**
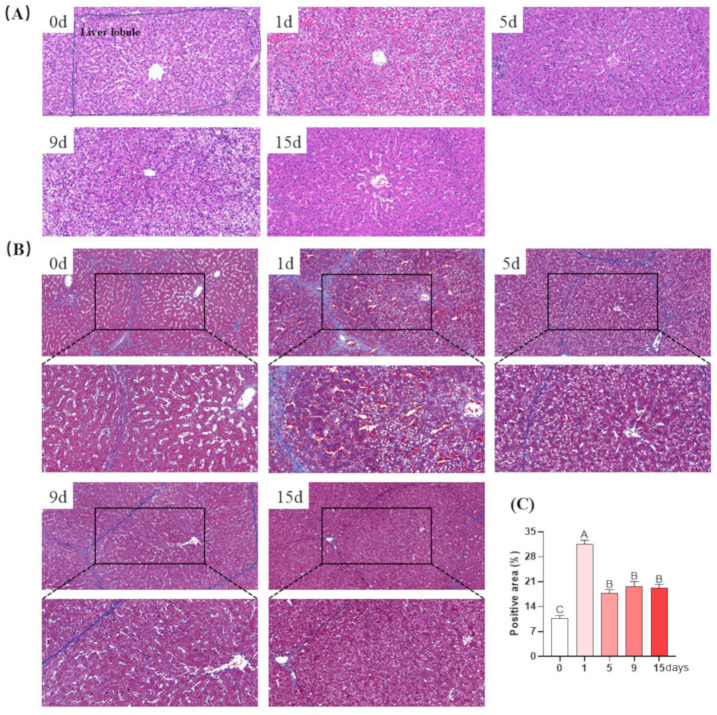
LPS-induced pathological liver changes in piglets. (**A**) Representative image of H&E staining in liver tissue (magnification 22×); (**B**) representative image of Masson staining in liver tissue (magnification 15×); (**C**) Masson staining semi-quantitative positive area for liver fibrosis. Values are the mean ± SEM (*n* = 8). The row values of different capital letters A, B, and C were significantly different (*p* < 0.05).

To evaluate the effects of LPS induction on liver damage and fibrosis, H&E and Masson’s trichromic stained sections were utilized (Figure 3). The liver morphology and structure of piglets on day 0 were normal, since the lobular structure was visible, the hepatocytes were arranged neatly, the nucleus was intact, and the cytoplasmic vacuoles were not obvious. On the first and fifth days of LPS injection, obvious damage to the liver was observed, as manifested by disordered hepatic cell cord arrangement, dissolved and vacuolized hepatocytes, karyopycnosis, and inflammatory cell infiltration and congestion. The abovementioned LPS injection-induced liver damage was attenuated on the ninth and fifteenth days (Figure 3A). 

The level of hepatic fibrosis was determined using Masson stain, as shown in Figure 3B. The livers of piglets in the day 0 group showed no blue collagen fibers. However, on the first and fifth days of LPS injection, the bile duct was greatly proliferated and collagen fibers strongly increased, causing fibrosis around the bile duct. On the ninth and fifteenth days, the LPS injection-induced hepatic fibrosis was assuaged, with only a small amount of proliferated bile ducts and increased collagen fibers.

### 3.3. Liver Inflammation

**Figure 4:**
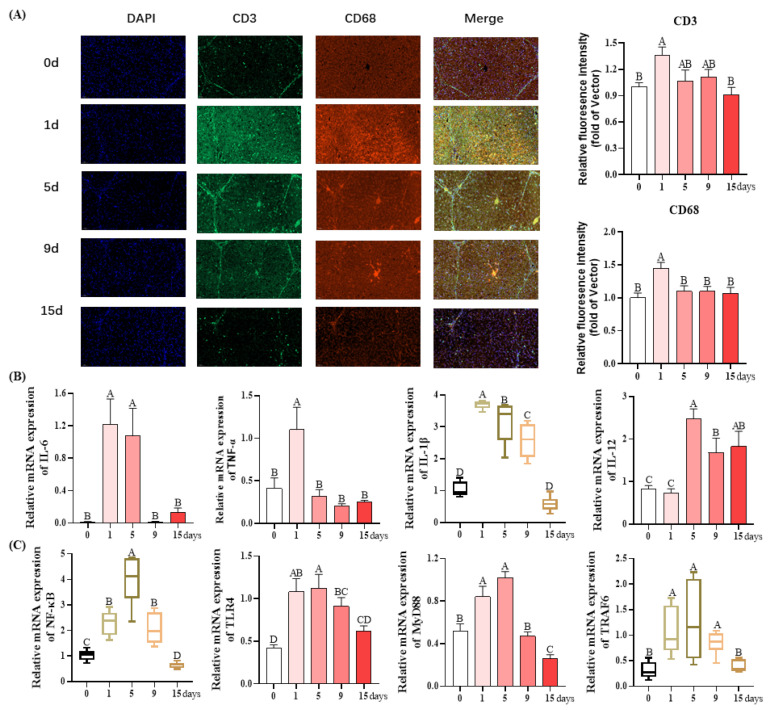
Inflammation of the liver under chronic immune stress induced by LPS. (**A**) Representative images and quantification of CD3^+^- and CD68^+^-positive cells. (**B**) The expression of inflammatory cytokines in liver. (**C**) The expression of TLR4 pathway in liver. Values are the mean ± SEM (*n* = 8). The row values of different capital letters A, B, C, and D were significantly different (*p* < 0.05). Data represented by the column bar graph were tested via one-way ANOVA, and data represented by the box plot were evaluated using the Welch test. IL-6, interleukin-6; TNF-α, tumor necrosis factor-α; IL-1β, interleukin-1β; IL-12, interleukin-12; NF-κB, nuclear factor kappa-B; TLR4, Toll-like receptor 4; MyD88, myeloid differentiation factor 88; TRAF6, TNF receptor-associated factor 6.

In the liver, both the quantification of CD3^+^- and CD68^+^-positive cells reached their highest values on day 1 of LPS injection (*p* < 0.05), and there were no differences among the other four groups (*p* < 0.05, Figure 4A). 

Then, the effects of chronic immune stress on the relative mRNA expression of inflammatory cytokines (IL-6, TNF-α, IL-1β, and IL-12) and genes (NF-κB, TLR4, MyD88, and TRFA6) in the liver were analyzed. As revealed in Figure 4B, significant increases in the mRNA expression levels of pro-inflammatory genes (IL-6, TNF-α, and IL-1β) were observed on day 1 compared with other groups, and all of them were restored to the similar value on day 15 as those seen on day 0 (*p* > 0.05). The mRNA expression of anti-inflammatory cytokine IL-12 was significantly elevated on day 5, 9, and 15 compared to day 0 in response to LPS injection (*p* > 0.05). 

As revealed in Figure 4C, compared with day 0, the NF-κB mRNA expression was significantly upregulated on day 1, 5, and 9, with the highest level observed on day 5 (*p* > 0.05) and was significantly downregulated on day 15 (*p* > 0.05). The mRNA expression levels of TLR4, MyD88, and TRAF6 were significantly elevated on day 1 and 5 relative to the day 0 (*p* > 0.05). No significant difference for TLR4 and TRAF6 mRNA expression was observed between day 0 and 15 (*p* > 0.05). The MyD88 mRNA expression was significantly downregulated on day 15 in comparison to day 0 (*p* > 0.05). 

### 3.4. Antioxidant and Oxidative Damage-Related Parameters of livers

**Figure 5:**
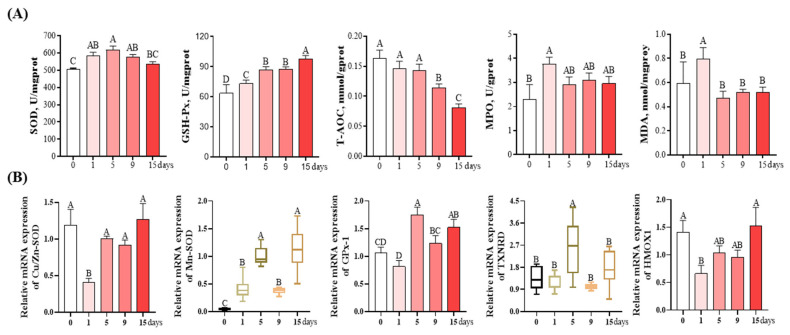
Changes in antioxidant capacity and oxidative damage parameters of the liver under chronic immune stress induced by LPS. (**A**) Changes in antioxidant enzyme activity and oxidative damage indexes in piglet liver. (**B**) Changes in the mRNA expression levels of oxidoreductase-related genes in piglet liver. Values are the mean ± SEM (*n* = 8). The row values of different capital letters A, B, C, and D were significantly different (*p* < 0.05). Data represented by the column bar graph were tested via one-way ANOVA, and the data represented by the box plot were evaluated via the Welch test. SOD, superoxide dismutase; GSH-Px, glutathione peroxidase; T-AOC, total antioxidant capacity; MPO, myeloperoxidase; MDA, malondialdehyde; Cu/Zn-SOD, copper and zinc superoxide dismutase; Mn-SOD, manganese-containing superoxide dismutase; GPx-1, glutathione peroxidase 1; TXNRD, thioredoxin reductase 1; HMOX1, heme oxygenase 1.

As shown in Figure 5A, compared to the day 0, the SOD activity was the lowest on day 0, gradually upregulated to its highest on day 5 (*p* < 0.05) and then showed a downregulation trend on day 9 and 15, although this was not statistically significant (*p* > 0.05). The activity of GSH-Px gradually increased from day 0 to 15 and reached its highest value on day 15 (*p* < 0.05). The activity of T-AOC on day 9 and 15 was significantly lower than that on other days (*p* < 0.05) and exhibited similar levels between these groups (*p* > 0.05). Compared to day 0, both the MPO and MDA content were significantly increased on day 1 (*p* < 0.05) and then showed a downtrend on days 5, 9, and 15; however, this was not of statistical significance (*p* > 0.05). 

As revealed in Figure 5B, compared to day 0, the mRNA expression of Cu/Zn-SOD on day 1 was significantly decreased by 66.39% (*p* < 0.05), and returned to a level similar as day 0 on subsequent days. The Mn-SOD mRNA expression was the lowest on day 0, and then significantly upregulated on other days, with the highest values observed on day 15 (*p* < 0.05). Compared with day 0, the GPx-1 mRNA expression showed a downward trend on day 1 and was significantly increased on other days, with the highest value observed on day 5 (*p* < 0.05). Compared with day 0, the mRNA expression of TXNRD increased by 88.97% on day 5 (*p* < 0.05), and no significant difference was observed between the other groups and the day 0 group (*p* > 0.05). The HMOX1 mRNA expression on day 1 was significantly downregulated by 53.19% compared to day 0 (*p* < 0.05), and gradually returned to a level similar as on day 0 over the next few days.

### 3.5. Mitochondrial Function of Livers

**Figure 6:**
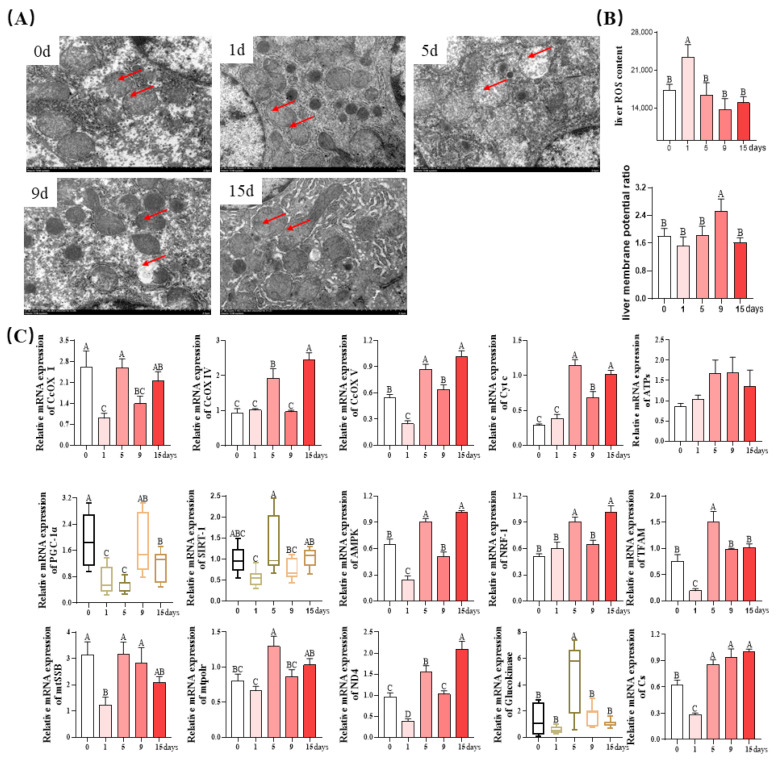
Liver mitochondrial morphology, biogenesis, and function under LPS-induced chronic immune stress: (**A**) Representative transmission electron microscopy images of liver mitochondria with arrows pointing to mitochondria; (**B**) Measurement of liver ROS content and membrane potential. (**C**) Quantification of mRNA expression of genes involved in mitochondrial function. Values are mean ± SEM (*n* = 8). The row values of different capital letters A, B, C, and D were significantly different (*p* < 0.05). Data represented by the column bar graph were tested via one-way ANOVA, and data represented by the box plot were evaluated by the Welch test. ROS, Reactive oxygen species; CcOX I, cytochrome c oxidase I; CcOX IV, cytochrome c oxidase IV; CcOX V, cytochrome c oxidase V; PGC-1α, peroxisome proliferator-activated receptor g coactivator-1; NRF-1, nuclear respiratory factor-1; TFAM, transcription factor a mitochondrial; SIRT-1, silent information regulator transcript 1; mtSSB, mitochondrial single-strand DNA-binding protein; mtpolr, mitochondrial RNA polymerase; ND4, NADH dehydrogenase subunit 4; CS, citrate synthase; AMPK, AMP-activated protein kinase; Cyt c, Cytochrome c; ATPs, ATP synthase.

As shown in Figure 6A, muscle mitochondria of piglets on days 0 and 15 showed a clearly defined internal membrane structure and wider cristae. On the first day of LPS injection, muscle mitochondria became expanded and vacuolated, and their membranes were dissolved. On the fifth day, very obvious mitochondrial swelling, sparse cristae, and vacuolization were observed. On the ninth day, the damage to liver mitochondria was largely alleviated, with only a small amount of mitochondrial swelling, sparse cristae, and vacuolization. In addition, the ROS production on day 1 was higher than that on other days (*p* < 0.05), and no significant difference was observed among these groups (*p* > 0.05, Figure 6B). The mitochondrial membrane potential ratio was significantly higher on day 9 than that on other days (*p* < 0.05), and no significant difference was observed among these groups (*p* > 0.05, Figure 6B).

Next, to explore the effects of chronic immune stress on mitochondrial function, the mRNA expression levels of mitochondrial biogenesis and function-related genes were determined. As shown in Figure 6C, compared with day 0, the mRNA expression of CcOX I, CcOX V, ND4, and Cs were significantly decreased on day 1 and increased on days 5 and 15 (*p* < 0.05). The mRNA expression of CcOX IV and Cyt c on days 5 and 15 was higher than that on day 0 (*p* < 0.05). While no significant differences were observed between the groups of ATPs mRNA expression, compared to day 0, the PGC-1α mRNA expression was significantly downregulated on days 1 and 5 (*p* < 0.05), and was restored to a similar value on day 9 as that of day 0. Compared to day 0, the SIRT-1 mRNA expression kept the same levels. However, after day 1 of LPS injection, the SIRT-1 mRNA expression increased dramatically on day 5 (*p* < 0.05) and was restored to a similar value on day 9 and day 15 as that seen on 0 (*p* > 0.05). Compared to day 0, the AMPK mRNA expression decreased dramatically by 62.5% on day 1 of LPS injection (*p* < 0.05), remained unchanged on day 9 (*p* > 0.05), and increased significantly on days 5 and 15 (*p* < 0.05). The NRF-1 mRNA expression on day 5 and 15 was of similar value and was significantly higher than that on days 0, 1, and 9 (*p* < 0.05), but no differences were detected among days 0, 1, and 9. Compared to day 0, the TFAM mRNA expression significantly decreased on day 1, increased on day 5 (*p* < 0.05), and remained unchanged on other days. The mtSSB mRNA expression on day 1 was lower than that on other days (*p* < 0.05). The mtpolr mRNA expression was the highest on day 5 and lowest on day 1, with intermediate values on other days (*p* < 0.05). The glucokinase mRNA expression on day 5 was highest, while the other days retained the same levels.

## 4. Discussion

Liver injury, fibrosis, and cirrhosis represent a major worldwide health problem. In the past few decades, accumulated scientific efforts have been made to further our understanding of these diseases. However, efforts to unravel the underlying molecular mechanisms of these diseases have encountered difficulties and discrepancies, possibly owing to the application of rodent models [35] or swine models of short term and acute stress [11,36]. Accordingly, an ideal swine model of long term and chronic stress which could advance our understanding of liver damage was thus certainly warranted. Blood biochemical indicators have been regarded as key indicators of body health and can reflect cell permeability and metabolic function [37]. In this study, the dynamic effects of chronic immune stress on liver injury-related enzyme activities in the sera of piglets were evaluated. Enzymes, such as AST, ALT, and ALP, are considered to be the main characteristic substances of liver injury, and an elevation in the serum activities of these enzymes suggests their leakage from damaged hepatocytes and is regarded as a useful quantitative marker of liver injury [38]. Serum CHE2 activity has been described as an indicator of cirrhosis [39]. In addition, serum TBA activities are positively correlated with liver fibrosis and cirrhosis and are markers of liver diseases [40]. Here, we found that LPS injection increased the activities of AST, ALT, ALP, CHE2, and TBA in the serum on day 1, but they were restored to similar values on day 15 as those seen on day 0, indicating that chronic immune stress induced liver damage in piglets in the early period (days 1~5). In agreement with the abovementioned results, liver morphology is compromised in the early period (days 1~5) under the influence of LPS and restored to normal values in the later period (days 9~15). These results are mostly in agreement with previous studies, which also showed that the serum AST activity of piglets was increased within 24 h of LPS injection [41].

Apart from its various metabolic functions, the liver is also an important immunological organ and enriched in natural killer, natural killer T cells, and macrophages (Kupffer cells) [42]. Since immune function exerts important roles in the development of liver injury, we next analyzed the expression of immune function-related parameters, such as CD3^+^ (T-cell marker) and CD68^+^ (macrophage-specific marker), via immunofluorescence [43,44]. The results showed that CD3^+^- and CD68^+^-positive proteins were significantly increased on the first day of LPS injection and, after day 5, returned to similar levels as those on day 0, which is in line with the alteration trend of blood biochemical indicators and liver morphology. These findings indicate that the incremental injection of LPS-induced injury and the repair of liver morphological could be modulated by the immune system. In acute or chronic liver diseases, Kupffer cells are able to release a variety of proinflammatory cytokines, such as IL-6, IL-1β, and TNF-α, or anti-inflammatory cytokines, such as IL-12, in the context of low physiological levels of LPS, which fulfill functions in the regulation of liver injury [45]. In agreement with the alteration trend of CD3 and CD68, the mRNA expression of IL-6, IL-1β, and TNF-α was significantly upregulated in the early phase (day 1), and were greatly reduced from day 5, and returned to the levels observed on day 0 in the later phase (day 15). In contrast, the mRNA expression of IL-12 was significantly upregulated in the later phase (days 5~15). In good accordance with our results, a large number of studies have reported that LPS-induced liver injury is associated with the massive expression of inflammatory factors, such as IL-6, TNF-α, IL-1β, and IL-12 [46,47,48,49]. Therefore, our results showed that in response to chronic immune stress, liver inflammation was caused in the early stage and attenuated in the later stage.

Given that inflammatory pathways in the liver are closely related to cytokines, we then measured the mRNA expression levels of TRAF6, MyD88, TLR4, and NF-κB [42]. As a member of the Toll-like protein family, TLR4 can be expressed in liver tissues and cells and can recognize LPS and mediate transmembrane signal transduction [50]. TLR4 activates MyD88 after sensing and binding to LPS outside the cell and further activates downstream molecules, such as TRAF6, eventually activating NF-κB [51]. The activation of NF-κB is able to promote the production of proinflammatory cytokines, which in turn activate the NF-κB pathway, thus forming a positive feedback regulation and amplifying the inflammatory reaction cascade [52]. Here, we found that the mRNA expression of TLR4, MyD88, TRAF6, and NF-κB were significantly upregulated in the early phase (day 1~5) and, in the later phase (day 9~15), were gradually restored to levels similar to those obtained on day 0, which was in good accordance with the histopathological alterations in liver morphology. These findings suggest that the repair of the injured liver morphology under the condition of chronic immune stress was related to the improved immune function and the downregulated activation of TLR4/MyD88 and NF-κB pathways.

Besides inflammation, oxidative stress is the other pathological process of liver injury. It has been reported that LPS exposure could cause oxidative stress, which is an important contributing factor to liver injury [53]. To explore the oxidative injury induced via the incremental injection of LPS, the content of MDA and MPO and the levels of antioxidant enzymes (such as SOD, GSH-Px, and T-AOC) in the liver of piglets were detected. Among these, SOD and GSH-Px are considered as the first line of defense of the antioxidant enzyme system against oxidative stress [54], and T-AOC reflects the cumulative effects of antioxidants in the body and serves as a comprehensive index for evaluating the antioxidant system [55]. All of these antioxidant enzymes can combat against liver injury caused by oxidative stress by eliminating ROS [25,56]. MPO, a heme-containing peroxidase, is highly expressed in multiple inflammatory cells, and MPO activation can catalyze the production of hypochlorous acid and other strong oxidants, thus triggering oxidative tissue damage and cell dysfunction and contributing to disease progression [57]. MDA is considered to be a key indicator of oxidative stress because it is the end product of lipid peroxidation [58]. In this study, we found that the liver activities of SOD and GSH-Px gradually elevated from day 0 to day 15 in response to LPS injection, whereas the liver activities of MPO and MDA increased in the early phase (days 1~5) and decreased in the later phase of LPS injection (days 5~15). These results were in parallel with the alteration trend of inflammatory cytokines. Moreover, the liver activities of SOD and GSH-Px followed time courses that were in line with the mRNA expression of Cu/Zn-SOD, Mn-SOD, and GPx-1. Both Cu/Zn-SOD and Mn-SOD were able to protect against oxidative damage by catalyzing the conversion of superoxide to oxygen and H_2_O_2_ [59,60]. As a major intracellular antioxidant enzyme, GPx-1 was able to catalyze the reduction of H_2_O_2_, resulting in the oxidation of glutathione [61]. Therefore, these findings indicate that lipid oxidative injury in the liver could be attenuated in the later period (days 5~15) of chronic immune stress, and the improved antioxidant capacity might be a contributing mechanism for reduced lipid oxidative injury.

Cellular mitochondria are implicated in a variety of physiological processes, including the cell death [62]. Several lines of evidence suggest mitochondrial involvement in the development of liver injury [63,64,65]. Moreover, mitochondrial function and oxidative stress are mechanistically interconnected [66]. In detail, high levels of oxidative stress can lead to mitochondrial dysfunction [67], disrupt electron transport and mitochondrial membrane potential, and impair ATP production, thus leading to cellular damage [68]. Therefore, we further investigated mitochondrial function-related parameters in the liver of chronic immune-stressed piglets. We found that the incremental injection of LPS greatly impaired the mitochondrial structure of the liver in the early phase of LPS injection (days 1~5), whereas the mitochondrial morphology gradually reverted to normal levels in the later phase (days 9~15). In agreement with the results of mitochondrial morphology, liver ROS content and the mRNA expression of electron transport strand-related genes (CcOX I, CcOX V, ND4, Glucokinase, and Cs) [69,70] as well as mitochondrial biogenesis-related genes (PGC-1α, TFAM, SIRT-1, mtSSB, mtpolr, and AMPK) [71,72] were significantly decreased on day 1 but reverted to similar values on day 15 as seen on day 0. Taken together, in response to chronic immune stress, the liver mitochondrial morphology and function were impaired in the early stage (days 1~5) and returned to normal levels in the later period (days 5~15).

In conclusion, we found that chronic immune stress, which was induced via the incremental injection of LPS impaired the liver morphology of weaned piglets and caused inflammation, oxidative stress, and mitochondrial dysfunction in the early phase (days 1~5). However, in the later phase (days 9~15), the liver morphology was gradually restored to normal, similar to that observed on day 0, and these effects could be associated with ameliorated inflammation and oxidative stress, as well as improved mitochondrial function. Our data indicate that weaned piglets underwent self-repair mechanisms in the liver in response to chronic immune stress. These findings provide insights into the dynamic changing pattern of the liver under the condition of chronic immune stress induced through the incremental injection of LPS and contribute to the development of effective therapies, which is especially helpful for clinical care directed to the management of liver damage of different origins. Despite these interesting observations, a number of questions remain unanswered. A key question is how the specific mechanism of liver chronic immune stress caused by incremental injection of LPS interacts with inflammation, oxidative stress, and mitochondrial function. Another important question is which nutrients can effectively alleviate liver damage and accelerate its repair. Further studies are undoubtedly warranted to resolve these problems.

## Figures and Tables

**Table 1:** The primers for RT-qPCR.

Genes	Sequence (5′ to 3′)	Size (bp)	Gene Bank No.
Interleukin-6, IL-6	F: AGCCCACCAGGAACGAAAGA	119	NM_214399.1
	R: AGCCATCACCAGAAGCAGCC		
Tumor necrosis factor-α, TNF-α	F: ATGGATGGGTGGATGAGAAA	129	NM_214022.1
	R: TGGAAACTGTTGGGGAGAAG		
Interleukin-12, IL-12	F: TTTCAGACCCGACGAACTCT	160	NM_214013
	R: CATTGGGGTACCAGTCCAAC		
Interleukin-1β, IL-1β	F: CAAAGGCCGCCAAGATATAA	147	NM_214055
	R: GAAATTCAGGCAGCAACAT		
Nuclear factor kappa-B, NF-κB	F: GACAACATCTCCTTGGCGGG	146	NM_001048232
	R: TCTGCTCCTGCTGCTTTGAGG		
Toll-like receptor 4, TLR4	F: TCAGTTCTCACCTTCCTCCTG	166	NM_001293316.1
	R: GTTCATTCCTCACCCAGTCTTC		
Myeloid differentiation factor 88, MyD88	F: GATGGTAGCGGTTGTCTCTGAT	148	NM_001099923.1
	R: GATGCTGGGGAACTCTTTCTTC		
TNF receptor associated factor 6, TRAF6	F: CAAGAGAATACCCAGTCGCACA	122	XM_013990069.2
	R: ATCCGAGACAAAGGGGAAGAA		
Copper and zinc superoxide dismutase, Cu/Zn-SOD	F: CAGGTCCTCACTTCAATCC	255	NM_001190422
	R: CCAAACGACTTCCASCAT		
Manganese-containing superoxide dismutase, Mn-SOD	F: GGACAAATCTGAGCCCTAACG	159	NM_214127
	R: CCTTGTTGAAACCGAGCC		
Glutathione peroxidase 1, GPx-1	F: TGGGGAGATCCTGAATTG	183	NM_214201
	R: GATAAACTTGGGGTCGGT		
Thioredoxin reductase 1, TXNRD	F: GTGCTGAGGAGCTTCCCGAGATGT	118	NM_214154.3
	R: TCCAGGACCATGACCCGCTTGTTAA		
Heme oxygenase 1, HMOX1	F: CGCTCCCGAATGAACACTCT	148	NM_001004027.1
	R: GCGAGGGTCTCTGGTCCTTA		
Peroxisome proliferator-activated receptor g coactivator-1α, PGC-1α	F: CCCGAAACAGTAGCAGAGACAAG	111	NM 213963
	R: CTGGGGTCAGAGGAAGAGATAAAG		
Nuclear respiratory factor-1, NRF-1	F: GCCAGTGAGATGAAGAGAAACG	166	AK237171.1
	R; CTACAGCAGGGACCAAAGTTCAC		
Transcription factor a mitochondrial, TFAM	F: GGTCCATCACAGGTAAAGCTGAA	167	AY923074.1
	R: ATAAGATCGTTTCGCCCAACTTC		
Silent information regulator transcript 1, Sirt-1	F: TGACTGTGAAGCTGTACGAGGAG	143	EU030283.2
	R: TGGCTCTATGAAACTGCTCTGG		
Mitochondrial single-strand DNA-binding protein, mtSSB	F: CTTTGAGGTAGTGCTGTGTCG	143	AK352341.1
	R: CTCACCCCTGACGATGAAGAC		
Mitochondrial RNA polymerase, mtpolr	F: CTTTGAGGTTTTCCAGCAGCAG	119	XM 001927064.1
	R: GCTCCCAGTTTTGGTTGACAG		
NADH dehydrogenase subunit 4, ND4	F: TTATTGGTGCCGGAGGTACTG	112	NM 001097468
	R: CCCAGTTTATTCCAGGGTTCTG		
Glucokinase	F: CTTTTCCCTCCCACACTGCTAT	119	AK233298.1
	R: GACTCCTCTTCCTGAGACCCTCT		
Citrate synthase, Cs	F: CCTTTCAGACCCCTACTTGTCCT	127	M21197.1
	R: CACATCTTTGCCGACTTCCTTC		
AMP-activated protein kinase, AMPK	F: ACCAGGACCCTTTGGCAGTT	100	NM_001167633.1
	R: GAATCAGGTGGGCTTGTTGC		
Cytochrome c oxidase I, CcOX Ⅰ	F: ATTATCCTGACGCATACACAGCA	127	AJ950517.1
	R: GCAGATACTTCTCGTTTTGATGC		
Cytochrome c oxidase Ⅳ, CcOX Ⅳ	F: CCAAGTGGGACTACGACAAGAAC	131	AK233334.1
	R: CCTGCTCGTTTATTAGCACTGG		
Cytochrome c oxidase Ⅳ, CcOX V	F: ATCTGGAGGTGGTGTTCCTACTG	160	AY786556.1
	R: GTTGGTGATGGAGGGGACTAAA		
Cytochrome c, Cyt c	F: TAGAAAAGGGAGGCAAACACAAG	154	NM 001129970.1
	R: GGATTCTCCAGGTACTCCATCAG		
ATP synthase, ATPs	F: TGTCCTCCTCCCTATCACACATT	116	AK230503
	R: TAGTGGTTATGACGTTGGCTTGA		
β-actin	F: TCTTTTCCAGCCTTCCTTCTTG	100	NM_007393
	R: GAGGTCTTTACGGATGTCAACG		

## Data Availability

Not applicable.
